# Signal transduction in photoreceptor histidine kinases

**DOI:** 10.1002/pro.3705

**Published:** 2019-08-20

**Authors:** Andreas Möglich

**Affiliations:** ^1^ Department of Biochemistry Universität Bayreuth Bayreuth Germany; ^2^ Bayreuth Center for Biochemistry & Molecular Biology Universität Bayreuth Bayreuth Germany; ^3^ North‐Bavarian NMR Center Universität Bayreuth Bayreuth Germany

**Keywords:** allostery, bacterial phytochrome, coiled coil, light‐oxygen‐voltage, sensor histidine kinase, sensory photoreceptor, sensory rhodopsin, signal transduction, two‐component system

## Abstract

Two‐component systems (TCS) constitute the predominant means by which prokaryotes read out and adapt to their environment. Canonical TCSs comprise a sensor histidine kinase (SHK), usually a transmembrane receptor, and a response regulator (RR). In signal‐dependent manner, the SHK autophosphorylates and in turn transfers the phosphoryl group to the RR which then elicits downstream responses, often in form of altered gene expression. SHKs also catalyze the hydrolysis of the phospho‐RR, hence, tightly adjusting the overall degree of RR phosphorylation. Photoreceptor histidine kinases are a subset of mostly soluble, cytosolic SHKs that sense light in the near‐ultraviolet to near‐infrared spectral range. Owing to their experimental tractability, photoreceptor histidine kinases serve as paradigms and provide unusually detailed molecular insight into signal detection, decoding, and regulation of SHK activity. The synthesis of recent results on receptors with light‐oxygen‐voltage, bacteriophytochrome and microbial rhodopsin sensor units identifies recurring, joint signaling strategies. Light signals are initially absorbed by the sensor module and converted into subtle rearrangements of α helices, mostly through pivoting and rotation. These conformational transitions propagate through parallel coiled‐coil linkers to the effector unit as changes in left‐handed superhelical winding. Within the effector, subtle conformations are triggered that modulate the solvent accessibility of residues engaged in the kinase and phosphatase activities. Taken together, a consistent view of the entire trajectory from signal detection to regulation of output emerges. The underlying allosteric mechanisms could widely apply to TCS signaling in general.

## 1 INTRODUCTION

**Figure 1:**
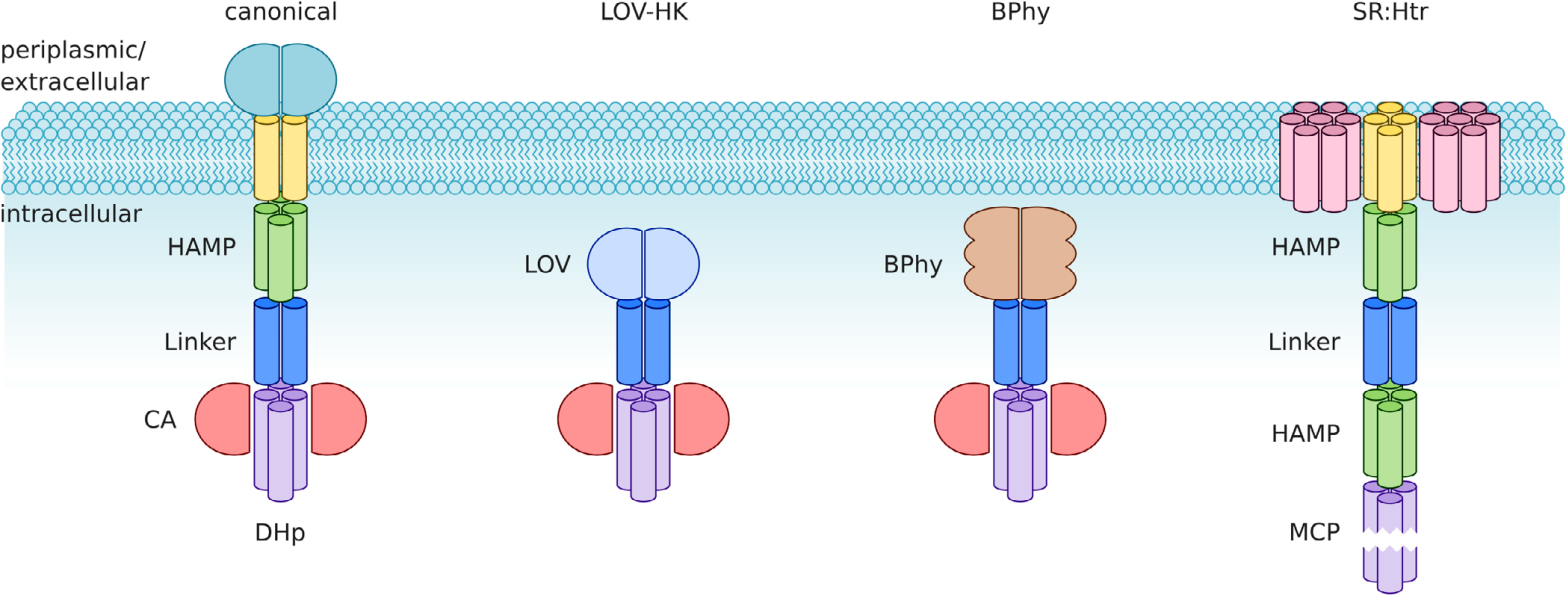
Protein architecture of sensor histidine kinases (SHK) and related receptors. Within canonical two‐component systems, the homodimeric SHK commonly spans the plasma membrane with extracellular/periplasmic sensor modules and intracellular effector modules. The transmembrane (TM) segment is formed by two parallel α helices and is often followed by a HAMP domain. A dimeric α‐helical coiled coil serves as the linker and connects to the effector, which comprises DHp and CA domains. Certain photoreceptor kinases, such as those with light‐oxygen‐voltage (LOV) or bacteriophytochrome (BPhy) sensors, are soluble, cytosolic proteins. They consequently lack the TM helices and the HAMP domain, thus showing much simpler architecture. Microbial rhodopsins can also serve as sensor modules for SHKs but the best‐studied photoreceptor of this class is sensory rhodopsin (SR) which forms a 2:2 complex with Htr, a methyl‐accepting chemotaxis protein (MCP). The SR:Htr complex shares with SHKs the homodimeric state, individual constituent domains and the overall architecture

Microorganisms commonly occupy habitats that are subject to frequent and profound fluctuations in conditions. To cope with a changing environment and to thereby ensure survival and eventual procreation, microorganisms must continuously read out their surroundings, process and integrate environmental signals, and decode these inputs into adequate cellular output. In many microorganisms, signal transduction is predominated by two‐component systems (TCS).^1^, ^2^, ^3^, ^4^, ^5^ TCSs mainly feature in prokaryotes and lower eukaryotes like yeast but also occur in higher plants. Canonical TCSs comprise a mostly homodimeric,^6^ transmembrane sensor histidine kinase (SHK) and a cytosolic response regulator (RR) (Figure 1). The sensor module of the SHK, commonly located in the extracellular room, the periplasmic space or inside the plasma membrane, modulates the enzymatic activity of the intracellular effector module. Said effector in turn consists of two segments, the all‐helical DHp domain (dimerization and phospho‐accepting histidine), and the catalytic (CA) domain. Despite sequence variations across different SHK families, the general architecture and arrangement of the DHp/CA effector is strikingly uniform which hints at overarching, joint signal transduction mechanisms. By contrast, SHKs employ a wide range of structurally and mechanistically disparate sensor modules. As a class, SHKs have evidently evolved to accommodate highly diverse sensory inputs and to channel them into a common regulatory output. Key to this remarkable convergence are α‐helical linker segments and domains that conjoin sensor and effector and transduce signals.

In signal‐dependent manner, SHKs autophosphorylate in *trans* or *cis* 7 at their eponymous histidine residues within the DHp domain, before relaying the phosphoryl moiety to aspartate sidechains of RRs. Once phosphorylated, the RR triggers downstream responses, which in many cases are of transcriptional nature and give rise to altered gene expression. The biological response to an environmental signal is thus effectively governed by the resultant phosphorylation level of the RR. Most SHKs not only catalyze the forward phosphorylation reaction but also the hydrolysis of the phospho‐aspartatyl anhydride in the phosphorylated RR.^8^, ^9^ It is hence the balance of the elementary kinase and phosphatase activities that determines the net output of the SHK and downstream responses for a given signaling state. By catalyzing the counteracting kinase and phosphatase reactions, SHKs realize fast and pronounced responses, which underpin the rapid and highly stringent adaptation of microorganisms to their environment.

**Figure 2:**
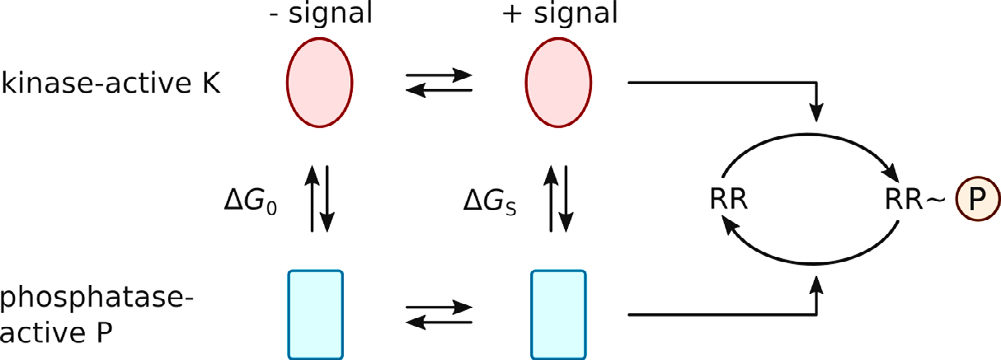
Allosteric model for signal transduction in sensor histidine kinases (SHK). The SHK is considered to exist in a dynamic equilibrium between a kinase‐active state K and a phosphatase‐active state P. The ratio of K over P is governed by the free energy difference between these states Δ*G*_0_ and Δ*G*_S_ in the presence and absence of signal. The kinase‐active state K promotes phosphorylation of the response regulator RR, and the phosphatase‐active P state catalyzes dephosphorylation. The physiological response is governed by the net phosphorylation state of the RR

The net activity of SHKs, that is, the balance between their kinase and phosphatase activities, can be recapitulated in a simple allosteric model that comprises two functional states existing in a signal‐dependent equilibrium,^10^, ^11^, ^12^, ^13^, ^14^ as determined by the free energy difference between these states (Figure 2). One state is distinguished by elementary kinase activity that outweighs the elementary phosphatase activity; hence, the net output is RR phosphorylation, and the state is denoted “kinase‐active state” (K). In the other state, the elementary phosphatase activity prevails, the net output is RR dephosphorylation, and the state is denoted “phosphatase‐active state” (P). Alternatively, the two states may be referred to as R/T (following classic allostery), on/off or hi/lo (in terms of net kinase activity), or asymmetric/symmetric (referring to the structure of the DHp/CA effector, see below). The basic allosteric model extends to other receptors, for example those engaged in chemotaxis, which share with TCSs certain types of sensor modules: in this case, the two states might be denoted as CW (kinase‐on)/CCW (kinase‐off), referring to whether they promote clockwise or counter‐clockwise flagellar beating. Likewise, the model could be expanded to consider additional states, for example, in case of receptors that sense and integrate multiple signals, see below.^15^ Notably, the model does not rule out the existence of additional states, nor does it posit that a given functional state would be associated with a unique structural state. In fact, it is evident that the effector modules of SHKs must adopt multiple conformations during autophosphorylation, phosphotransfer to the RR and dephosphorylation. A core tenet of allostery is that the functional (and structural) states are inherent to the receptor and encoded in its amino acid sequence. Put another way, the effector has a propensity to assume certain states. Regulation is allosteric in that the presence of signal merely shifts the equilibrium between states, that is, it changes the free energy difference between them, but does not alter their molecular structure per se. These fundamental considerations already go a long way toward accounting for the diversity of sensor modules among TCSs. Even structurally disparate inputs can be accommodated as long as they lead to allosteric modulation between the K and P functional states of the SHK. In focusing on photoreceptor histidine kinases, belonging to a group of TCSs that respond to light, this review addresses how input signals are decoded into shifting the equilibrium between these functional states that determine the net output activity of the SHK.

## 2 PHOTORECEPTOR HISTIDINE KINASES

Sensory photoreceptors enable sensation of light and underlie central organismal processes, for example, phototaxis, vision, and development. Light is generally absorbed by a chromophore embedded within the photosensor module of the photoreceptor. Depending upon chromophore type and the photochemical reaction sequence triggered upon light absorption, sensory photoreceptors divide into around 10 distinct classes.^16^, ^17^ Among these classes, light‐oxygen‐voltage (LOV) receptors,^18^, ^19^ bacterial phytochromes (BPhy)^20^, ^21^ and microbial rhodopsins (Rho)^22^ are particularly relevant for the present scope, as they recur as sensor modules to SHKs. Notably, BLUF^23^ and cyanobacteriochrome^21^, ^24^, ^25^ sensor units are also found as constituent parts of SHKs but their structural and mechanistic characterization lags that in the other three classes. Photoreceptor histidine kinases have been serving as paradigms for TCS signaling as they afford a number of advantages. The study of conventional, light‐inert SHKs is complicated by the input signal whose nature is often unknown at the molecular level or which exhibits limited tractability, for example, in case of temperature‐responsive SHKs. By contrast, in photoreceptor histidine kinases the molecular identity of the input, that is, light, is known, and it can be applied or withdrawn with ease and speed, thereby enabling comparatively straight‐forward analyses in both the presence and absence of signal, and both at steady state and in time‐resolved manner. As visible light penetrates cell walls and membranes, most photoreceptor classes, and in particular LOV and BPhy receptors, are cytosolic, soluble proteins which greatly facilitates structural, biophysical, and mechanistic studies, even in the context of the full‐length receptor. Conversely, with certain exceptions,^26^ light‐inert SHKs are predominantly transmembrane proteins that decode extracellular or periplasmic inputs into intracellular output. To date, transmembrane SHKs have eluded structure determination at full length. Structural data on SHKs are hence routinely acquired for protein fragments, often bearing mutations that predispose the receptor fragment toward a certain functional state.

For the stated reasons, photoreceptor histidine kinases have provided unusually detailed molecular views of signal transduction in SHKs. The comparative analysis of recent findings on receptors with LOV, BPhy and Rho sensor units pinpoints recurring themes and converging mechanisms that may widely apply to light‐sensitive SHKs and, by extension, to TCSs in general. At the same time, photoreceptor histidine kinases have found ample use in optogenetics^27^ where they enable the specific, spatiotemporally acute, and reversible control by light of microbial state and physiology.^28^, ^29^, ^30^ In successive sections, this review treats the molecular decoding of photosensory input, its transduction through α‐helical linker elements, and the resultant modulation of effector output.

## 3 PHOTOSENSORY INPUT

As motivated above, this review focuses on light‐sensitive SHKs that employ LOV, BPhy, and Rho sensor units. The following subsections consider for each unit how light absorption by the dark‐adapted resting state is converted into conformational changes within the photosensor that can propagate downstream to the effector module.

### 3.1 *Light‐oxygen‐voltage photosensors*

**Figure 3:**
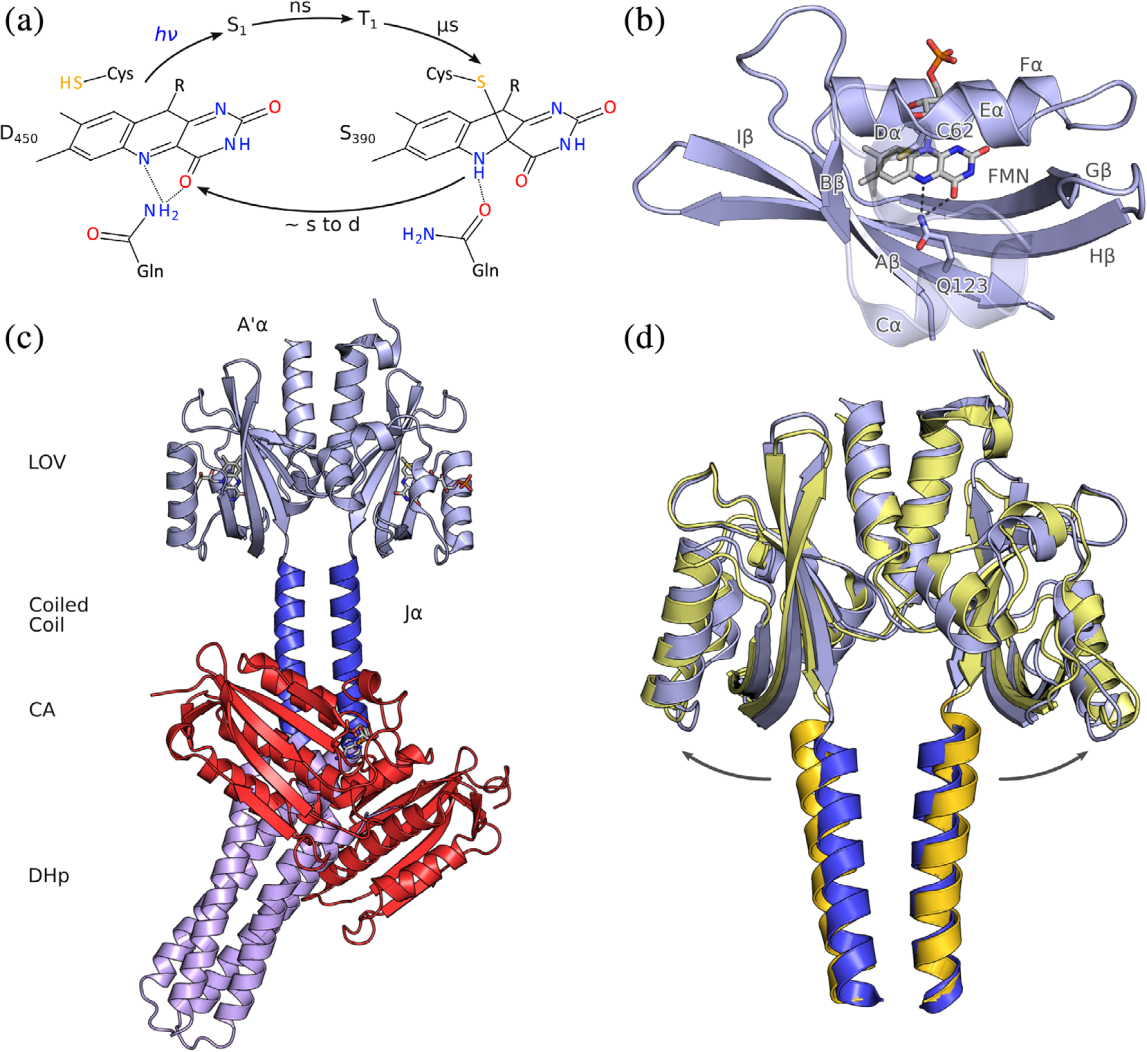
Photochemistry, structure, and signaling of light‐oxygen‐voltage (LOV)‐histidine kinases. (a) The dark‐adapted state D_450_ with its flavin‐nucleotide chromophore in the oxidized quinone state absorbs blue light and then passes through short‐lived electronically excited singlet and triplet states. A covalent bonds forms between a conserved cysteine residue and atom C4a of the flavin, thus giving rise to the metastable signaling state S_390_, which thermally decays to D_450_ over seconds to many hours, depending on residues adjacent to the chromophore. (b), As a subfamily of the Per‐ARNT‐Sim domains, LOV sensor domains exhibit a compact fold with a five‐stranded antiparallel β sheet and several α helices that together coordinate the flavin chromophore. (c), The full‐length structure of the dark‐adapted LOV histidine kinase YF1 (PDB 4GCZ). (d), Based on electron paramagnetic resonance spectroscopy and X‐ray solution scattering, the light‐induced conformational changes within the dimeric LOV sensor have been identified as a splaying apart of the sister monomers. The N termini of the Jα helices that form the coiled‐coil linker are thus moved apart by around 3 å in the light (yellow) relative to the dark (blue)

Originally identified as the blue‐light‐responsive photosensor modules of plant phototropins,^18^, ^31^ light‐oxygen‐voltage (LOV) domains also recur in many prokaryotes.^32^, ^33^ The first bacterial LOV receptor to be characterized in detail has been YtvA from *Bacillus subtilis* (*Bs*YtvA) which triggers the general stress response as a function of light.^32^, ^34^, ^35^ Through phylogenetic analyses, LOV proteins have since been identified in diverse organisms and architectural contexts,^36^ with histidine kinases representing the most frequent effector module of prokaryotic LOV receptors.^36^ Other common effectors in prokaryotic LOV receptors include DNA‐binding domains and GGDEF enzymes that catalyze the production of the bacterial second messenger cyclic diguanylate. With few exceptions,^37^, ^38^ the LOV photosensor is N‐terminal of the effector, usually separated by a short, mostly α‐helical linker element. By contrast, the so‐called short LOV proteins lack a covalently attached effector and presumably transduce signals in *trans* to a distinct protein. LOV photosensors absorb light in the UV‐A to blue range of the electromagnetic spectrum via a non‐covalently bound flavin‐nucleotide chromophore, most often flavin mononucleotide (FMN) but in some cases^39^ flavin adenine dinucleotide (FAD) (Figure 3a). In the absence of light, the flavin chromophore usually resides in its oxidized quinone state, denoted D_450_, but certain LOV receptors may undergo chemical reduction under physiological conditions and may hence serve as biological sensors for redox potential.^39^, ^40^ To the extent it has been investigated, the redox midpoint potential for flavins embedded in LOV proteins ranges from around −260 to −320 mV^40^, ^41^ which is only slightly more negative than the intracellular reduction potential reported for *Escherichia coli* 42 and mammalian cells.^43^ Depending on cellular context, LOV receptors may thus exist as a mixture of their oxidized quinone and more reduced semiquinone and hydroquinone forms. Within the canonical LOV photocycle, absorption of UV‐A/blue light by the D_450_ quinone electronically excites the flavin to S_1_ or higher singlet states, followed by efficient inter‐system crossing on the nanosecond time scale to the triplet state T_1_. The triplet state decays to the signaling state S_390_ on the microsecond timescale via formation of a covalent thioether between atom C4a of the flavin isoalloxazine ring and atom Sγ of an adjacent, conserved cysteine residue within the LOV photosensor.^44^ Bond formation likely proceeds via a neutral radical‐pair intermediate^45^, ^46^ and leads to protonation of the flavin N5 atom. As a consequence, a conserved glutamine residue in hydrogen‐bonding contact with N5 undergoes a 180° flip of its amide side chain which triggers further rearrangements in hydrogen bonding throughout the LOV photosensor, see below. The signaling state is metastable and recovers to the dark‐adapted state in a base‐catalyzed process^47^ with kinetics governed by temperature, solvent composition^47^ and molecular environment^48^, ^49^ of the flavin chromophore. If the conserved cysteine is removed by mutagenesis, LOV photosensors display photoreduction to the neutral semiquinone state (NSQ) which also possesses a protonated N5 atom.^50^, ^51^, ^52^ As recently demonstrated, the NSQ state shows downstream signaling responses akin to those elicited by the thioadduct state, thus conclusively demonstrating that N5 protonation is both necessary and sufficient for LOV signal transduction^53^; bond strain, altered electronic environment and a slight tilt of the flavin ring observed in the thioadduct state are apparently dispensable. This view is borne out in LOV photosensors reconstituted with 5‐deazaflavin nucleotides,^54^ which can still form a covalent thioadduct upon illumination but are incapable of eliciting downstream signaling processes, arguably owing to the absence of a protonable group at the flavin 5 position. Notably, flavin reduction can also be accomplished by chemical means, raising the possibility that LOV photosensors have arisen from ancestral precursors engaged in the sensing of redox potential or oxygen.^53^ As a corollary, flavin‐based sensors for these disparate stimuli potentially employ closely similar signal transduction strategies.

#### 3.1.1 *LOV structure*

LOV photosensors form a subclass of the widespread Per‐ARNT‐Sim domain family, members of which serve as versatile interaction and sensing modules.^12^ The first structure of an isolated LOV domain,^55^ that of LOV2 from *Adiantum capillus‐veneris* phototropin 1, already elucidated the essential features of the core photosensor domain that are present in all LOV structures determined since. In particular, the flavin nucleotide chromophore is embedded in a cavity formed by a five‐stranded antiparallel β‐sheet (Aβ‐Bβ‐Gβ‐Hβ‐Iβ) and by helices Eα and Fα (Figure 3b). The polar pterin moiety of the flavin is coordinated by several amide side chains, and the apolar dimethyl benzene moiety forms van‐der‐Waals interactions with mostly aliphatic residues. The conserved cysteine residue, located in helix Eα, is poised above the plane of the flavin ring system, and the conserved glutamine residue resides in strand Iβ, directly juxtaposed to the flavin N5 atom. It has become ever more apparent that the functional LOV photosensor extends beyond the PAS core domain, as for example defined by Pfam,^56^ and also encompasses N‐ and C‐terminal extensions which are mostly α‐helical in conformation and are denoted as A′α and Jα, respectively. The structural diversity of these ancillary elements contrasts with the largely invariant LOV core, as do the variable quaternary arrangements evidenced in LOV receptors. In the paradigmatic plant phototropin LOV photosensors, first to be characterized in molecular detail, the A′α and Jα helices pack onto the outer face of the PAS β‐sheet.^57^, ^58^ At least as isolated domains, phototropin LOV photosensors are monomeric and respond to blue‐light illumination with reversible unfolding and detachment of Jα^57^ and likely also of A′α.^59^ LOV photosensors attached to histidine kinases feature a markedly different architecture, arguably best exemplified in the three‐dimensional structure of the engineered, blue‐light‐inhibited SHK YF1^60^ (Figure 3c). Although YF1 represents a chimera^61^ between the *Bs*YtvA LOV sensor module and the effector module of FixL from *Bradyrhizobium diazoefficiens* 62 (reclassified from *Bradyrhizobium japonicum* 63), it is prototypic for the architecture of naturally occurring SHKs.^56^, ^61^, ^64^ In darkness, YF1 readily phosphorylates its cognate response regulator FixJ from *B. diazoefficiens* but under blue light YF1 is converted into a net phosphatase that actively removes the phosphoryl group from phospho‐FixJ. Within YF1, two LOV photosensors associate into a parallel homodimer with the interface composed of the outer faces of the β‐sheets and a short, parallel coiled coil formed by the N‐terminal A′α appendices. Notably, the A′α helices predominantly undergo intermolecular interactions with the opposite monomer and thereby interlock the two LOV photosensors. The C‐terminal Jα helices, which directly feed into the antiparallel DHp four‐helix bundle and thereby furnish the connection to the effector module, assemble into a second coiled coil that is coaxial with the one formed by the A′α helices.

#### 3.1.2 *LOV signaling*

A large body of recent functional and structural data on YF1, related LOV receptors and SHKs have let emerge a consistent and unprecedentedly detailed molecular picture of signal detection and transduction. In the dark‐adapted state of LOV receptors, the conserved glutamine residue forms hydrogen bonds via its amide ε‐NH_2_ group to atoms N5 and O4 of the flavin isoalloxazine ring. As described above, light‐induced protonation of the flavin at atom N5, be it via formation of the covalent thioadduct,^44^ be it by photoreduction,^53^ constitutes the key event in forming the signaling state of LOV receptors and in triggering downstream responses. To satisfy hydrogen bonding in the signaling state, the glutamine undergoes a 180° flip^65^ such that its amide ε‐O atom interacts with the newly protonated N5. As a result, the amide ε‐NH_2_ group points away from the flavin ring and is left to enter new hydrogen‐bonding interactions. A molecular dynamics study^66^ on the *Neurospora crassa* Vivid (*Nc*VVD) protein has provided a precise molecular view of subsequent events that is fully consistent with the experimental characterization of this LOV receptor.^39^, ^67^ In the simulations of the light‐adapted thioadduct and photoreduced states, the ε‐NH_2_ group of the glutamine (residue Q182 in *Nc*VVD) enters a new hydrogen bond with the backbone carbonyl O atom of A72 which is located at the start of strand Aβ. Formation of this bond bestows local stability on this region but concomitantly weakens the interaction with the N‐terminal appendix of *Nc*VVD, referred to as the N‐cap. Resultant refolding of the N‐cap allows *Nc*VVD to adopt a homodimeric state, which is crucial for downstream signaling. Evidence for phototropin LOV photosensors implicates that upon flipping, the conserved glutamine engages in new hydrogen bonds with the hydrophilic side chains or the backbone of structurally equivalent residues at the start of Aβ.^65^, ^68^ In general, for different LOV receptors these initial light‐induced conformational changes consistently culminate in a destabilization of the interaction between the outer face of the β‐sheet and N‐ and C‐terminal ancillary elements packed against it, often causing their detachment. This is most prominently evidenced in the dissociation and unfolding of the Jα helix in phototropin LOV sensors^57^ but is also reflected in the DNA‐binding LOV receptors aureochromes^69^, ^70^ and EL222,^71^ *Neurospora crassa* Vivid,^39^, ^67^ RGS‐LOV proteins,^72^ a recently discovered RNA‐binding LOV receptor,^38^ and in the monomeric LOV sensor histidine kinase EL346.^6^

Applied to YF1 as a paradigm for the more prevalent dimeric SHKs, the following scenario emerges. Protonation of flavin N5 triggers flipping of glutamine 123 which could then engage in a new hydrogen bond with the carbonyl O of glycine 26 that is structurally equivalent to A72 in *Nc*VVD.^60^, ^73^ Although still awaiting in‐depth structural characterization, subtle rearrangements of Aβ and the adjacent Iβ strand that harbors Q123 likely result and weaken the interaction with the A′α helix of the sister LOV photosensor. Due to the entangling of the A′α helices, complete dissociation of the LOV monomers is prevented. As recently demonstrated,^74^, ^75^, ^76^ the weakening of the β‐sheet:A′α interaction is rather channeled into a slight rotation and tilting apart of the monomers (Figure 3d). This quaternary transition entails a separation of the Jα anchor sites at the tips of the Iβ strands by around 3 å which provides the mechanistic basis for signal propagation to the linker and the histidine kinase effector unit, see below. The structural changes manifest within the LOV sensor after blue‐light absorption in a single concerted step on the microsecond timescale, synchronously with thioadduct formation. Notably, consistent light‐induced conformational transitions have been observed by two independent experimental techniques (double electron–electron resonance [DEER]^74^ spectroscopy and X‐ray solution scattering^75^, ^76^), and in the context of either the isolated *Bs*YtvA photosensor or the composite LOV‐SHK YF1. These findings not only confirm the observed signaling mode, but also they imply that this mode is little affected by C‐terminal appendage of an effector module. Indeed, similar mechanisms are likely at play in other prokaryotic LOV receptors. As a case in point, *Pp*SB1 from *Pseudomonas putida* that belongs to the class of short‐LOV proteins lacking covalently attached effector modules has been crystallized in both its dark‐adapted^77^ and light‐adapted states.^78^ Overall, the structure of *Pp*SB1 resembles that of the YF1 photosensor with two LOV monomers associating in parallel orientation via their β‐sheets and the A′α coiled coil. The comparison of the two *Pp*SB1 crystal structures indicates that in the light‐adapted state one LOV monomer is rotated with respect to the other by around 29°, accompanied by an increase in the distance of separation between the Jα anchor sites at the end of Iβ by 3.8 å relative to the dark‐adapted state. These structural and mechanistic similarities indicate that the principal signaling mode is widely shared across homodimeric PAS and LOV receptors. The functional importance of the β‐sheet:A′α interface as a crucial hub for signal processing and transduction is underlined by mutational studies on several PAS receptors where residue exchanges in this region prompted altered effector output.^79^, ^80^, ^81^, ^82^, ^83^ As a case in point, certain exchanges of single residues at the β‐sheet:A′α interface sufficed for inversion of the signal response of YF1 to blue light.^79^

Taken together, the paradigm YF1 illustrates how electromagnetic waves are absorbed as a signal and converted into quaternary structural changes within the sensor that generate a simple output: the moving apart of the Jα linker helices at their bases. The amplitude of the structural change may appear small at first glance but is on the same scale as that observed in certain chemoreceptors.^84^ Similar signaling mechanisms likely exist in other, light‐inert receptors, as indicated by the exchangeability of chemosensor and photosensor modules,^61^ and by the multiple roles flavin nucleotides can assume as cofactors in the sensing of light, oxygen and redox potential.^53^

### 3.2 *Bacteriophytochromes*

**Figure 4:**
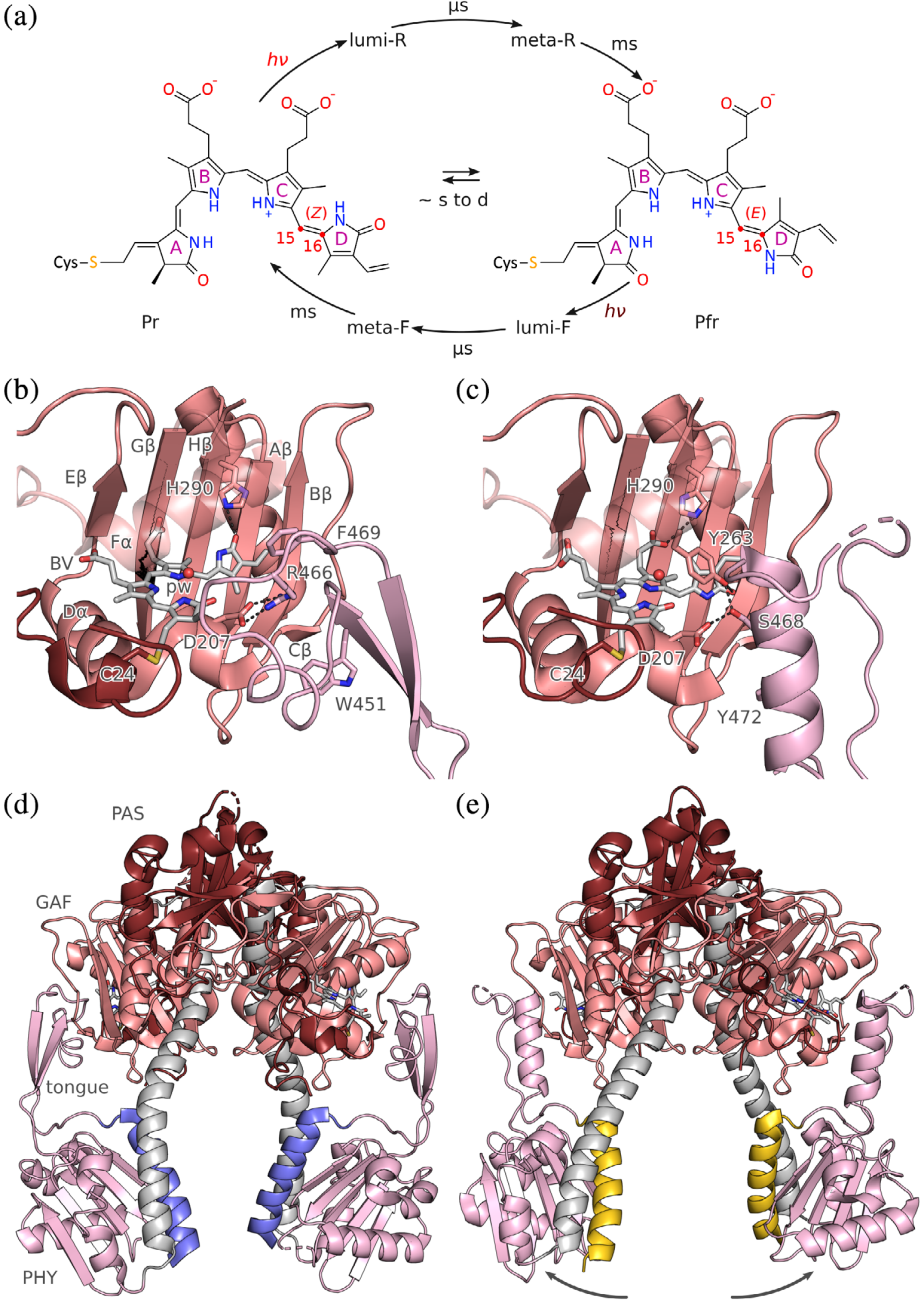
Photochemistry, structure and signaling of bacteriophytochrome (BPhy)‐histidine kinases. (a) Phytochromes adopt two (metastable) states that absorb red and far‐red light, respectively, and are hence denoted Pr and Pfr. The Pr state is characterized by a *Z* configuration of the bilin chromophore around its C15 = C16 double bond, and the Pfr state by an *E* configuration. Red and far‐red light drive the Pr → Pfr and Pfr → Pr transitions, both of which proceed through short‐lived excited intermediates. The thermal recovery between Pr and Pfr is usually slow. (b) Configuration of the chromophore‐binding pocket within the GAF domain in the Pr state of *Deinococcus radiodurans* BPhy (PDB 4Q0J). The biliverdin (BV) chromophore in its 15*Z* conformation is coordinated by several sidechains and a conserved water molecule (denoted pw, pyrrole water), and the PHY tongue assumes a β conformation (see main text). (c) As in panel B but for the Pfr state with the BV in the 15*E* configuration and the tongue in α conformation (5C5K). (d) Stucture of the *D. radiodurans* PAS‐GAF‐PHY photosensory core module in the Pr state. The output helices that transition into the ensuing coiled‐coil linker are marked in blue. (e) As in panel D but for the Pfr state. The connector helix (grey) has straightened, thereby pulling apart the PHY domains and the output helices (yellow)

Phytochromes (Phy)^20^, ^21^, ^85^ are the photoreceptors responsible for a series of red‐light‐dependent physiological adaptations in higher plants, for example, the onset of flowering and germination.^86^, ^87^ Early spectroscopic analyses of plant shoots revealed phytochromes to harbor a pigment that can be photochromically switched between red‐absorbing (Pr, *λ*_max_ ≈ 660 nm) and far‐red‐absorbing (Pfr, *λ*_max_ ≈ 730 nm) states.^88^ Aided by sequence homology, phytochromes were also discovered in both photosynthetic and non‐photosynthetic prokaryotes.^89^, ^90^, ^91^ With few exceptions, the photosensors of Phys generally comprise consecutive PAS, GAF, and PHY domains which together are denoted as the “photosensory (core) module” (PSM or PCM). In plant Phys, the effector module is a histidine‐kinase‐related domain that possesses sequence homology to SHKs but lacks catalytically important residues, including the eponymous histidine. Bacterial Phys display a range of different effector entities whose activity is regulated in light‐dependent manner, most often histidine kinases as well as cyclases (GGDEF) and phosphodiesterases (EAL) for the second messenger cyclic di‐guanosine monophosphate. Embedded in their GAF domains, Phys bind as chromophores linear tetrapyrroles (or, bilins) that are derived from heme by oxidative cleavage and are covalently attached to a cysteine residue as a thioether. Whereas the bacterial phytochromes, referred to as bacteriophytochromes (BPhy) in the following, incorporate biliverdin (BV), cyanobacterial and plant phytochromes make use of the more reduced bilins phycocyanobilin (PCB) and phytochromobilin (PФB), respectively. The more extended conjugated π electron system in BV relative to PCB/PФB and the attachment to different C atoms within the bilin moiety (to the vinyl 3^2^ carbon for BV, and to the 3^1^ carbon for PCB/PФB) cause a red shift by about 30–40 nm of the Pr and Pfr absorption maxima in BPhys. Structural investigation of BPhys and plant Phys (see below) revealed a largely planar arrangement of the four pyrrole rings A–D which contrasts with the helical conformation assumed by isolated bilins in solution.^92^ In the red‐absorbing Pr state the bilin adopts the 5*Z*_syn_, 10*Z*_syn_, 15*Z*_anti_ (*ZZZ*ssa) configuration, and in Pfr it adopts the (*ZZE*ssa) configuration (Figure 4a). For conventional Phys, the Pr state is the thermodynamically more stable state that prevails in the dark; by contrast, the so‐called bathyphytochromes feature Pfr as the dark‐adapted state. It is not yet fully understood which sequence and structural determinants govern the nature of the dark‐adapted state. Red and far‐red light drive the *Z → E* and *E → Z* isomerization, respectively, of the bilin D ring around the C15 = C16 bond. Both isomerization reactions proceed via short‐lived intermediates denoted lumi‐R and metaR, or lumi‐F and meta‐F, respectively. Complete *Z* ↔ *E* photoisomerization requires the presence of all three domains that constitute the PCM; in particular, removal of the PHY domain leads to incomplete *Z*/*E* isomerization. Notably, D‐ring isomerization represents the key event in the Phy photocycle, and downstream signal propagation is thus abolished in the absence of PHY. Interestingly, cyanobacteriochromes (CBCR)^21^, ^24^, ^25^ also utilize bilin chromophores and closely related photochemistry, yet they are realized as stand‐alone GAF domains and are thus evidently capable of signal transduction in the absence of PHY. In addition, CBCRs display a number of mechanisms, for example, formation of a second thioether bond between a cysteine residue and the C10 atom of the bilin, to diversify their photochemistry which can greatly differ from the 15*Z*/Pr:15*E*/Pfr photocycle of conventional Phys.^21^, ^24^, ^25^ Similar mechanisms of spectral tuning are at play in a lineage of algal Phys despite them possessing a PAS‐GAF‐PHY scaffold,^93^, ^94^ thus suggesting that similar photochemical diversity may exist in other bacterial and plant Phys or might be obtainable via protein engineering.

#### 3.2.1 *Bacteriophytochrome structure*

The advent of bacterial Phys has greatly eased sample preparation and thus paved the way to highly resolved structural studies.^95^, ^96^, ^97^, ^98^ Following the initial elucidation of the PAS‐GAF tandem of *Deinococcus radiodurans* BPhy (*Dr*BPhy), several structures of complete PCMs (i.e., PAS‐GAF‐PHY) of BPhys have yielded detailed information covering both the Pr and Pfr states and thereby greatly informing on the mechanisms of photoreception and signal transduction. In particular, the PCM of *Dr*BPhy has been structurally resolved in both its dark‐adapted Pr and the illuminated Pfr states.^99^, ^100^ Irrespective of state, the three globular domains PAS, GAF, and PHY adopt highly similar folds characterized by a central β sheet and several α helices.^101^ Whereas the PAS and GAF domains are in immediate contact, the PHY domain is held at a distance by a long connector helix. A prominent protrusion, denoted tongue, emanates from the PHY domain to interact with the GAF domain and its embedded bilin chromophore, an intramolecular contact that is essential for signal transduction, see below. The very N terminus of the PAS domain threads through a loop connecting the PAS and GAF domains and thereby forms a knot in the structure. The tertiary structure of different PCMs is well conserved with low pairwise root‐mean‐square displacement values. A notable exception is the spatial arrangement of the C‐terminal PHY domain and the connector helix. Especially among the Pr structures, and to lesser extent among the Pfr structures, different orientations are observed. To accommodate these arrangements, the long connector helix can bend at a defined hinge point in its middle.^92^, ^99^, ^102^ This conserved position represents an intended weak link in the connector and is of key functional relevance for signal transduction, see below. The BPhy PCM structures usually reveal a homodimeric arrangement with the interface formed by the long connector helix and several shorter helices belonging to the GAF and PHY domains. In most cases, the dimer has parallel (or, head‐to‐head) orientation but antiparallel homodimers are also present.^103^ As the PCMs have mostly been structurally elucidated as truncated proteins without attached effector modules, the observed quaternary structure need not necessarily correspond to the one in the full‐length receptor. The parallel arrangement appears physiologically more relevant as it is supported by structural data on BPhys with attached effector modules^104^, ^105^, ^106^, ^107^ and by the parallel homodimeric nature of the most common BPhy effector modules, see above. Notably, the architecture of the parallel PCM homodimer is reminiscent of the LOV‐SHK architecture^60^ that also features parallel homodimers, α‐helical interface and laterally suspended globular sensor domains of the PAS superfamily.

The bilin chromophore of Phys is coordinated inside the GAF domain via polar and nonpolar interactions with amino acid side chains and ordered water molecules (Figure 4b). In addition, the vinyl substituent of ring A forms a thioether to a cysteine residue that is located in the N‐terminal extension of the PAS domain in case of BPhys or within helix Fα of the GAF domain in case of plant Phys. Whereas the pyrrole rings A–C are roughly coplanar, ring D is tilted out of the plane in both the Pr and Pfr structures (Figure 4b,c). Commensurate with its essential function in signal transduction, the bilin forms contacts with all three domains that make up the PCM.

#### 3.2.2 *Bacteriophytochrome signaling*

A wealth of structural data on the Pr and Pfr states of complete PCMs, in certain cases both the Pr and Pfr forms of the same BPhy, has delivered a detailed understanding of signal‐transduction processes. Using the arguably best‐characterized BPhy, that from *D. radiodurans*, as a paradigm, the series of structural events that underpin the transition from the dark‐adapted Pr state to the Pfr signaling state are summarized. For a more detailed description, the reader is referred to References 99 and 100. Within Pr, the BV chromophore is stabilized in its 15*Z* form by a hydrogen bond of ring D with the conserved histidine 290 situated in strand Hβ of the GAF domain (Figure 4b); interestingly, this position structurally corresponds to that occupied by the conserved glutamine residue in LOV photosensors, see above. The PHY tongue forms a β‐hairpin docked against the GAF domain and stabilized by interactions of R466, part of the conserved ^465^PRXSF^469^ motif within the PHY tongue, with D207 and Y263 within the GAF core. Additional interactions between the PHY tongue and the GAF core are mediated by F469 and W451, the latter of which is situated within the conserved W^G^/_A_G motif.^108^ Light‐induced *Z*/*E* isomerization of the BV chromophore triggers a series of conformational rearrangements that propagate throughout the entire PCM (Figure 4c). In a mechanism called “flip‐and‐rotate,” 109 isomerization of the D ring is accompanied by a slight rotation of the bilin chromophore around an axis perpendicular to the planes of rings B and C. Reorientation of the D ring triggers changes in the conformation and interaction of residue side chains within the chromophore‐binding pocket. In particular, the salt bridge D207:R466 is broken, thus leaving D207 free to engage in a new hydrogen bond with S468 located in the PRXSF motif. Residue Y263 which in Pr also interacted with R466 now stabilizes the 15*E* conformation of BV by hydrogen‐bonding to the carbonyl group of the D ring. To accommodate these new interactions, the PHY tongue undergoes substantive refolding from β‐hairpin to α‐helix conformation, accompanied by the so‐called tryptophan switch^108^: W451 within the W^G^/_A_G motif is displaced from a hydrophobic pocket on the surface of the GAF core by residue Y472 within the conserved WXE motif (in most BPhys a tryptophan rather than tyrosine is found in the W position). The transition from the extended β conformation to α conformation causes a compaction and shortening of the PHY tongue. Therefore, the PHY domains are pulled apart, and the long connector helices, which are kinked in the Pr state, straighten out around the hinge region in the connector helix (Figure 4d,e). Crucially, the entire C‐terminal halves of the PCM, comprising half of the long connector as well as PHY core and tongue, move concertedly as a rigid body. Therefore, the very C‐terminal helices of the PCM that directly transition into the linker helices and from there into the DHp effector module exactly trace these movements, thus achieving downstream propagation of the signal.

The available Pr and Pfr PCM structures, including those of bathy‐BPhys and *A. thaliana* PhyB, largely conform to the scenario laid out for *Dr*BPhy. Comparative analyses by X‐ray solution scattering revealed that the light‐induced structural changes within the PCM associated with the Pr → Pfr transition are similar across several BPhys.^110^ Hence, it appears likely that the above signaling mechanism generally holds for Phys although it is open to which extent bathy and plant Phys differ. With but few exceptions, the PCMs have been structurally elucidated in isolation, that is, as PAS‐GAF‐PHY constructs without covalently attached C‐terminal effector module. Where present in the structure,^104^, ^105^, ^107^, ^111^ the effectors are mostly connected to the PCM via a continuous parallel coiled coil, thus possibly restricting the quaternary structures the PCM can adopt in the Pr and Pfr states. In particular, it remains to be seen whether the large‐scale splaying apart of the PHY domains observed for the isolated *Dr*BPhy PCM manifests to the same extent within the context of full‐length native and engineered BPhy receptors.^105^, ^111^, ^112^, ^113^, ^114^, ^115^ In fact, recent structures of a BPhy‐GGDEF enzyme indicate that the conformational transitions within the context of a full‐length receptor are of much smaller amplitude.^107^ Regardless, the long continuous α helices are conducive to downstream relay of the conformational signal, see below.

### 3.3 *Sensory rhodopsins*

As extensively reviewed elsewhere,^22^, ^116^, ^117^ rhodopsin photoreceptors consist of an opsin apoprotein and a covalently bound retinal chromophore. In contrast to the above LOV and phytochrome photoreceptors, rhodopsins are not soluble but integral membrane proteins. Rhodopsins divide into two principal classes: Microbial or type‐I rhodopsins comprise a diverse and growing group that serve as light‐driven proton and ion pumps (e.g., bacteriorhodopsin [BR] and halorhodopsin [HR]), as light‐gated proton and ion channels (channelrhodopsin [ChR]), or as sensor modules for receptors with enzymatic output.^118^ By contrast, animal or type‐II rhodopsins generally function as G‐protein coupled receptors (GPCRs) and are involved in vision and photoentrainment of the circadian clock. The group of enzyme‐associated microbial rhodopsins includes members that control the activity of histidine kinases, as exemplified by sensory rhodopsins (SR). Originally, two homologous SRs, denoted SRI and SRII, were discovered in the halophilic *Halobacterium salinarium* where they mediate positive (i.e., attractive light signals) and negative (i.e., repellent light signals) phototaxis. Downstream transduction of light signals is accomplished by interactions of *Hs*SRI and *Hs*SRII with their cognate transducer proteins called *Hs*HtrI and *Hs*HtrII, respectively. The C‐terminal portions of the Htr transducers harbor MCP (methyl‐accepting chemotaxis protein) domains, which form signaling complexes with components of the chemotactic protein machinery.^119^ In response to absorption of repellent blue light by *Hs*SRII, a phospho‐relay cascade is triggered which culminates in phosphorylation of the response regulator CheY. Phospho‐CheY in turn promotes clock‐wise (CW) rotation of the flagellar motor which results in a tumbling motion and negative phototaxis of *H. salinarium*. Conversely, in case of *Hs*SRI, orange light serves as an attractant and ultimately induces counterclockwise (CCW) flagellar rotation, smooth swimming, and positive phototaxis of the bacterium. Interestingly, the subsequent absorption of UV light switches the *Hs*SRI:*Hs*HtrI complex such that it mediates CW flagellar rotation and negative phototaxis. Whereas SRs are indirectly coupled to histidine kinases via the Htr transducers, several rhodopsins are covalently linked to histidine kinase effectors, for example HKR1 from *Chlamydomonas reinhardtii*.^120^ *Cr*HKR1 and related rhodopsins with covalently attached nucleotide cyclase^121^ and phosphodiesterase effectors^118^, ^122^ are subsumed as enzymerhodopsins.^123^ As functional data on enzymerhodopsins are sparse and no structural information is available yet, the focus of the present discussion will be on SR. However, enzymerhodopsins and *Cr*HKR1 in particular arguably represent integrated versions of the SR:Htr complex and may well employ signal transduction mechanisms related to the one detailed for SR below. This notion is further supported by the finding that a covalent fusion between SRII from *Natronomonas pharaonis* (*Np*SRII) and its HtrII partner (*Np*HtrII) supports intact phototaxis *H*. s*alinarium* 124; intriguingly, the general architecture of enzymerhodopisns with covalently linked effectors was thus effectively anticipated before their actual discovery.

**Figure 5:**
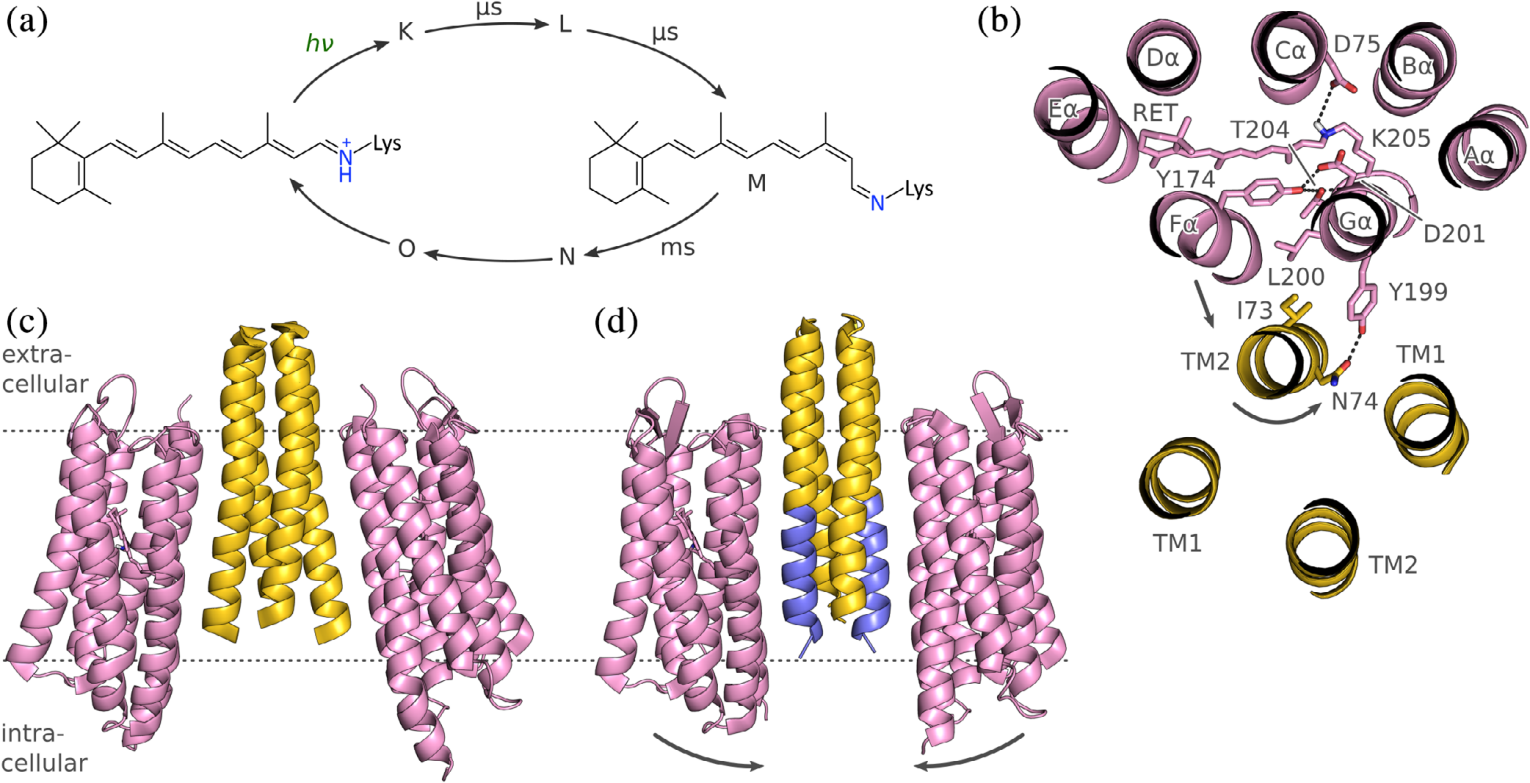
Photochemistry, structure and signaling of sensory rhodopsin (SR). (a) Microbial rhodopsins harness the fully reversible all‐*trans* to 13‐*cis* isomerization of a retinal chromophore, bound to a lysine residue as a protonated retinal Schiff base (RSB). Light absorption triggers a photocycle that comprises several short‐lived intermediates before and after the signaling state M in which the RSB is deprotonated. Note that the photocycle intermediates are based on the bacteriorhodopsin nomenclature,^22^ and the time constants refer to the overall reaction sequence from the dark‐adapted to the M state and vice versa. (b) Rhodopsins possess seven transmembrane helices. Sensory rhodopsin II from *Natronomonas pharaonis* (PDB 1H2S), viewed from the extracellular side, forms a 2:2 complex with its transducer HtrII that in turn consists of two helices TM1 and TM2 (for clarity, only one SR molecule is shown). Residues highlighted in sticks are thought to be instrumental in transducing light signals. Upon light absorption, the helix Fα tilts outward and thereby prompts a counter‐clockwise rotation of TM2 (when regarded from the extracellular), accompanied by a slight piston motion. (c) Overall structure of the SRII:HtrII complex from *N. pharaonis* in the V shape. (d) A different crystal form (PDB 5JJE) shows an altered U shape of the complex in which the TM2 helices (blue) run nearly parallel. The two complex orientations could reflect genuine states assumed during signal transduction and might be of functional relevance (see main text)

The retinal chromophore of rhodopsins is bound to a conserved lysine residue as an imine that is commonly referred to as the retinal Schiff base (RSB) and that is generally protonated in the dark‐adapted state (Figure 5a). Whereas microbial rhodopsins undergo photoisomerization of their retinal chromophores from the all‐*trans* to the 13‐*cis* form, animal rhodopsins employ 11‐*cis* to all‐*trans* photoisomerization. In their dark‐adapted states, microbial rhodopsins typically absorb light between 520 and 580 nm, and animal rhodopsins between 480 and 525 nm.^22^ However, as not least evident in vertebrate vision, rhodopsins display a broad range of color sensitivities, much more diverse than for LOV receptors, which are effectively restricted to the blue spectral range. Color sensitivity is determined by the energy gap between the S_0_ ground and S_1_ excited electronic states, and color tuning can accordingly be effected by (de)stabilization of either or both states (for an excellent treatise, see Reference 22). Principal factors affecting color sensitivity of rhodopsins are the protonation state of the RSB; polar and electrostatic interactions with protein residues, in particular with the so‐called counterion (see below) near the RSB; and the degree of conjugation in the retinal π electron system as governed by the geometry and planarity of the chromophore, specifically of the retinal β‐ionone ring. Although these factors are well understood and can be recapitulated in molecular simulations, the deliberate color tuning remains challenging, given that any residue exchange must not interfere with the function of a given rhodopsin. This is especially true if large spectral shifts are sought that would require simultaneous exchange of several residues.

Photon absorption initiates the photocycle of microbial rhodopsins that has been extensively studied, not least owing to a large body of experiments on BR, the archetypical microbial rhodopsin.^22^ Sensory rhodopsins display an overall similar photocycle as BR, and the same is likely true for enzymerhodopsins as well. Briefly, upon photoexcitation the microbial rhodopsin photoreceptor undergoes fast bond isomerization on the picosecond timescale to the 13‐*cis* form, and a series of structural intermediates, denoted K, L, M, N, and O, are consecutively populated. In certain rhodopsins, additional intermediates are observed, for example, the M_1_ and M_2_ states in the photocycle of *N. pharaonis* SRII.^116^ Whereas the photocycle in BR and HR is completed within ~10 ms to allow for rapid and repetitive ion pumping under high‐light conditions, the photocycle of SR is much longer to enable sensitive light perception even under low‐light conditions. Within the M state, the RSB is deprotonated, which represents a key event for eliciting downstream signal transduction, see below. All photocycle intermediates possess an RSB in the 13‐*cis* form except for the final O state, which has returned to the all‐*trans* conformation. Because the O state thermally reverts to the dark‐adapted state and thus completes the photocycle, enzymatic regeneration of the retinal chromophore as occurs in animal rhodopsins is obsolete for microbial rhodopsins. The M through O intermediates are considered the signaling states of SR, and accordingly these states are relatively long‐lived and persist for milliseconds to even seconds. Due to their long lifetime, certain intermediates display photochromic quality in that absorption of a second photon drives conversion to a different state, in some cases abridging the photocycle. For example, in *Hs*SRI absorption of a first photon around 580 nm leads to population of the M state that triggers positive chemotaxis; subsequent absorption of UV light by the M state drives conversion to another state, denoted P_520_, that mediates negative chemotaxis (see above) and that eventually reverts to the dark‐adapted state in thermal manner.^116^

#### 3.3.1 *Sensory rhodopsin structure*

Rhodopsins are representatives of the widespread family of 7‐helix transmembrane (7TM) receptors and comprise helices Aα, Bα, Cα, Dα, Eα, Fα, and Gα, each of which traverses the plasma membrane once (Figure 5b). Generally, the N terminus is outside of the cell but recent evidence suggests the presence of a prepended, additional transmembrane helix in a rhodopsin guanylate cyclase (RhoGC) which would put the N terminus of this particular receptor intracellularly.^125^ The C terminus is generally inside the cell thus allowing covalent attachment of cytosolic effector modules in case of the enzymerhodopsins, such as *Cr*HKR1 and RhoGC. The present discussion of structural aspects and signaling processes focuses on the best studied sensory rhodopsin *Np*SRII from *N. pharaonis*, and below residue numbers refer to this specific photoreceptor. In *Np*SRII, the retinal chromophore forms a protonated Schiff base with the conserved lysine 205 situated in the terminal helix Gα. The isoprene tail and the β‐ionone ring of the retinal are embedded between hydrophobic residues located in helices Cα–Fα. Aspartate 75 in helix Cα serves as the counterion to the positively charged, protonated RSB. In immediate vicinity of the RSB, the sidechains of Y174 (in helix Fα) and of T204 (Gα) form a hydrogen‐bonding network with the backbone carbonyl oxygens of L200 and D201 (both in Gα). The N‐terminal portion of the transducer *Np*HtrII forms two membrane‐spanning helices, denoted TM1 and TM2, that associate with the outer faces of helices Fα and Gα of *Np*SRII. This complex is stabilized by hydrophobic interactions and two clusters of polar interactions, one formed by T189 (Gα), E43 (TM1) and S62 (TM2), and the other formed by Y199 (Gα) and N74 (TM2). Within the membrane, *Np*SRII forms a complex with the cognate transducer *Np*HtrII in 2:2 stoichiometry, thus recapitulating the general C_2_‐symmetric architecture of conventional SHKs with an α‐helical spine along the symmetry axis and laterally appended sensor units. The C‐terminal segment of *Np*HtrII has not been structurally resolved but sequence homology^56^ indicates that TM2 directly feeds into the parallel four‐helix bundle of a HAMP domain, see below.

#### 3.3.2 *Sensory rhodopsin signaling*

The light‐induced all‐*trans* to 13‐*cis* isomerization as part of the transition from the dark‐adapted state to the K state triggers conformational rearrangements within the opsin protein that manifest at different stages in the photocycle, especially upon entering the M state. Inspection of the structures of the ground and M states reveal that these rearrangements are surprisingly subtle, at least when assessed by the conventional approach of freeze trapping within the crystal lattice. Particularly, the *trans*/*cis* (or, *E*/*Z*) isomerization of the C13 = C14 retinal double bond is compensated by rotation around single bonds in the lysine sidechain of the RSB, and the displacement of individual atoms is thus spatially limited. Of central importance, in the 13‐*cis* conformation the hydrogen substituent of atom C14 points toward and sterically interferes with the hydrogen‐bonding network formed by Y174, L200, D201, and T204, see above. Transitions within this network lead to an outward tilt of helix Fα, as detected by electron paramagnetic resonance (EPR) spectroscopy.^126^, ^127^ In addition, TM2 rotates by around 20–30° and undergoes a piston motion relative to the plane of the membrane which together entails a weakening of the SRII:HtrII interaction.^127^ As a corollary, the HAMP domain of HtrII is thought to dissociate from SRII.

A challenge in fully understanding the SR signal trajectory arises from the minute amplitude of light‐induced structural changes in the crystal lattice, which are much smaller than those detected by complementary experimental techniques such as EPR. Moreover, the structural differences between crystal forms of the *Np*SRII:*Np*HtrII complex much exceed those induced by light within a given, single crystal form. Specifically, in the original structure of the 2:2 complex the two SR monomers adopt a V shape where the halves of SR that point toward the intracellular are further apart than those pointing toward the exterior (Figure 5c). More recently, a U shape of the complex was resolved in which the SR monomers are in near parallel orientation (Figure 5d). The difference in inclination relative to the plane of the plasma membrane averages 8° and is maximal for helix Gα with around 11°.^128^ Although the functional relevance of the two shapes is not yet clear, it is tempting to speculate that they represent two snapshots of the signal trajectory. According to this view, the U shape could reflect the dark‐adapted state and the V shape the signaling M state. The U‐to‐V transition could facilitate signal transduction to the HAMP domain of *Np*HtrII, which is in dynamic equilibrium between different conformational states, see below. Support for this scenario derives from a molecular dynamics study which proposed highly similar quaternary structural rearrangements even prior to publication of the U‐shape structure of the *Np*SRII:*Np*HtrII complex.^129^

As pointed out above, enzymerhodopsins can be considered integrated versions of the SR:Htr complex. It is tantalizing to speculate that key elements of the above signal transduction mechanism are also realized in enzymerhodopsins. However, the verification or invalidation of this hypothesis awaits the detailed molecular and structural characterization of these photoreceptors.

## 4 SIGNAL TRANSMISSION THROUGH α‐HELICAL BUNDLES AND LINKERS

The above chapters reveal the rich diversity and ingenuity of structural mechanisms by which the information content of incident light is processed. The disparity of the underlying photosensing mechanisms contrasts with an astounding convergence at the level of the simple, structural output generated by the sensor unit: the principal mode of downstream signal transmission appears to be helical displacements, chiefly tilting (or, pivoting) and rotation within α‐helical bundles. The recurring output mode explains why even structurally disparate sensors and effectors can productively interact with another and why sensors can often be functionally exchanged between different receptors. Similarly, the underlying mechanistic principles likely pertain to considerable extent to other effector classes as well. As a case in point, structures of the nitrate/nitrite‐sensing histidine kinase NarQ from *Escherichia coli* 130 illustrate signal‐induced pivoting of α helices and their translocation relative to the membrane. This chapter explores how helical rearrangements are transmitted through linkers to the effector module of photoreceptor histidine kinases.

### 4.1 *The structure of α‐helical coiled coils*

**Figure 6:**
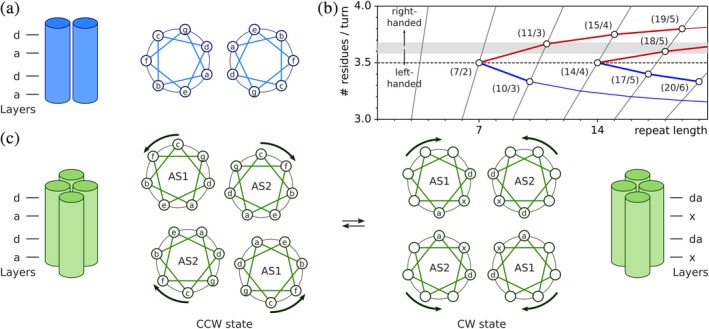
Coiled‐coil architecture. (a) The parallel homodimeric coiled coil is formed by two α‐helices that run in register in the same direction and are wound around each other. Coiled coils are characterized by a periodic sequence pattern, which in case of the regular parallel coiled coil, covers seven residues, denoted *a*–*g*, and two α‐helical turns. The positions *a* and *d* are preferentially occupied by hydrophobic residues and stabilize the coiled coil. (b) In addition to the heptad periodicity examined in panel A with seven residues per two helical turns (7/2), parallel coiled coils can assume a range of other periodic assemblies that are characterized by their repeat lengths and number of residues per turn. Coiled coils with more than ~3.6 residues per turn exhibit right‐handed superhelical winding, and those with fewer show left‐handed superhelical winding. The figure is based on Lupas and Gruber^134^ and drawn with fit‐o‐mat.^170^ (c) HAMP domains form a parallel four‐helical bundle with the helices named AS1 and AS2. HAMP domains are in dynamic equilibrium between two states, referred to as CCW and CW, that differ in the packing and angular orientation of the helices. The arrows indicate concerted helical rotations of AS1 and AS2 that convert the CCW into the CW state and vice versa. The figure is based on Sukomon et al.^143^

There is overwhelming evidence at the structural and sequence levels that the linker segments connecting the sensor and effector moieties of SHKs (that is, at least of the canonical dimeric specimens) generally form parallel α‐helical coiled coils. Often, the linker helix is also referred to as the signaling or S helix.^131^ As summarized in excellent articles on the topic, coiled coils are architecturally well‐understood^132^, ^133^, ^134^ to the extent that their structures can be predicted with high levels of confidence.^135^ Given the preeminence of coiled coils in SHK signaling, a brief discussion in the present context is nonetheless warranted; for an authoritative, detailed treatise, the reader is referred to pertinent reviews.^132^, ^133^, ^134^ In coiled coils, two or more α‐helices are in register and are wound around a common central (or, superhelical) axis, either in parallel or antiparallel orientation. The resultant helical assembly is stabilized by knobs‐into‐holes packing where sidechains of one helix (i.e., the “knobs”) periodically protrude into depressions (i.e., the “holes”) formed by sidechains of the partner helix or helices, respectively (Figure 6A). By contrast, interactions between α‐helices in non‐coiled‐coil assemblies are mostly out of register and utilize ridges‐into‐grooves packing modes. Coiled coils are found in multiple architectures with greatly varying length, stoichiometry, and topology of the constituent helices. Three types of coiled coil are particularly relevant for SHKs, that is, the parallel homodimeric (A_2_), the antiparallel heterotetrameric ($A_{2}{\bar{B}}_{2}$), and the parallel heterotetrameric (A_2_B_2_) forms which, respectively, feature in the linker, the effector DHp and HAMP domains. As discussed below, HAMP domains frequently occur as insertions in the linkers of many transmembrane SHKs.

Coiled coils are stabilized by interactions between residues that periodically repeat along the constituent helices. An integer number of residues *m* makes up an integer number *n* of α‐helical turns relative to the common central axis; the homodimeric parallel coiled coil A_2_ features a so‐called heptad repeat in that two (*n* = 2) such turns are formed by seven residues (*m* = 7) (Figure 6B). However, the ideal geometry of the unperturbed right‐handed α helix is characterized by around 3.6 residues per one turn which is close to yet slightly different from the number of 7/2 = 3.5 residues per turn for a helix in the A_2_ coiled coil. If the two α helices in a coiled coil ran exactly parallel to each other within a plane, a slight overwinding would thus be imposed on them (i.e., fewer than 3.6 residues per turn). As originally postulated by Crick,^136^ the seemingly conflicting structural requirements are reconciled by supercoiling of the coiled coil; that is, the central axis of the coiled coil is not a straight line but a helix in itself. (Notably, similar general concepts apply to DNA topology where partial unwinding of the right‐handed B‐form DNA can be compensated by right‐handed [or, negative] supercoiling.^137^) The directionality and degree of supercoiling directly result from satisfying both the *m*/*n* periodicity of the coiled coil and the 3.6 residues per turn of unperturbed α helices; for A_2_, left‐handed supercoiling, that is, the opposite handedness to the right‐handed α helices, is obtained. The left‐handed A_2_ supercoil is characterized by the length along the central axis over which one superhelical turn is completed, denoted pitch *P*, of ~140 å and by the angle at which the constituent helices are inclined relative to another, denoted helix‐crossing, or interface angle, of ~20°. In the case of two α helices that run parallel to each other, that is, that do not exhibit any supercoiling, the crossing angle is 0°.

By convention, the residue positions of A_2_ are periodically labeled with lower case letters *a* through *g*, where residues *a* and *d* denote positions at the interface of the coiled coil that are predominantly hydrophobic.^138^ This geometric arrangement gives rise to knobs‐into‐holes packing (also denoted *a*–*d* packing) and alternating residue layers at the coiled‐coil interface. Within the *a* layer, the side chain of residue *a* protrudes into a pocket formed by four residues of the juxtaposed helix, namely *a* and *g* of the same layer and two residues *d* in the layers below and above; within the *d* layer, the interactions are with *d* and *e* of the same layer and with two residues *a* in the layers below and above. Deviations from the regular heptad succession of residues *a*–*g* abound and give rise to structural alterations of the coiled coil. The addition of one residue is called a skip. The insertion of three residues is referred to as a stammer, and results in a 10/3 coiled coil which has more pronounced left‐handed supercoiling than the 7/2 form (Figure 6B). In general, with more pronounced supercoiling, the helix‐crossing angle asymptotically approaches 90°, where the limit value corresponds to the physically nonsensical scenario of infinite supercoiling. For example, within the 10/3 form, the crossing angle between the helices is between 40 and 50°. In contrast to the stammer, the insertion of four residues into the heptad repeat, denoted a stutter, results in a 11/3 coiled coil, that is, one with ~3.67 residues per turn, and promotes right‐handed supercoiling. All other deletions/insertions into the heptad register can be expressed as a combination of stammers and stutters, sometimes delocalized over several α‐helical turns. A stutter can for example give rise to an 18/5 rather than an 11/3 coiled coil.^134^ Deviations from the 7/2 geometry are accommodated by different packing modes, with local adoption of complementary *x*‐*da* geometry and knobs‐to‐knobs packing of side chains. Within a *da* layer, the residues *a* and *d* from both helices form a ring around the central A_2_ axis; in an *x* layer, two residues point directly at another across the central axis.

Higher‐order coiled coils, including A_2_B_2_ and $A_{2}{\bar{B}}_{2}$, also employ knobs‐into‐holes packing and resultant *a* and *d* layers. Often, the higher‐order assembly is additionally mediated by an extended hydrophobic core, formed by seams of hydrophobic residues running down the individual helices. In tetrameric coiled coils, these seams usually overlap by one residue position, for example, *g* + *d* and *d* + *a* with a shared *d* position, or *d* + *a* and *a* + *e* with a shared *a* position. As for A_2_, deviations from knobs‐into‐holes packing and its *d* and *a* layers occur frequently in multimeric coiled coils as well, mainly in antiparallel assemblies. Unusually for a parallel‐coiled coil, HAMP domains which are of the heterotetrameric A_2_B_2_ type can also assume packing modes that deviate from the canonical knobs‐into‐holes *a*–*d* packing. Within the obligate homodimeric HAMP domains, the first α helix, termed AS1 or α1, terminates in a short connector that loops back and leads to the second helix AS2 (or, α2) (Figure 6C). Beyond this common architecture, individual structures of HAMP domains exhibit a variety of subtly different conformations and helical packing modes.^139^, ^140^, ^141^, ^142^ Despite these differences, the known HAMP structures can be assigned to two principal classes that are often referred to as the CW and CCW states, based on their frequent occurrence in chemotaxis receptors where they turn on kinase activity, promoting clockwise (CW) flagellar beating, or turn it off, promoting CCW beating, respectively.^141^, ^143^ The first reported HAMP structure^139^ adopted the CW state, in which the A_2_B_2_ coiled coil has complementary *x*‐*da* packing of the helices. As explained above, within alternating layers residues either point at another across the coiled‐coil axis (*x* layer) or form a hydrophobic ring around this axis (*da* layer). Other HAMP structures elucidated the CCW state that exhibits conventional *a*–*d*‐type knobs‐into‐holes packing. Within the CCW state, the AS2 helices associate closely and effectively form a two‐helix bundle, whereas the AS1 helices move somewhat out from the coiled‐coil axis. Within the dynamic‐bundle model, the CW and CCW states are also ascribed looser or tighter packing, respectively.^144^, ^145^ A third state, denoted CCW(B) can be assumed by certain HAMP domains but is unstable, and hence, its biological relevance remains unclear. Comparative structural analyses of the CW and CCW states identify systematic differences in the position and orientation of the AS1 and AS2 helices. Between the CW and CCW states, the helices are rotated around their axes by around 360°/7/2 ≈ 26°, often accompanied by changes in their crossing angles and more subtle conformational transitions (Figure 6C). By embedding a given HAMP domain into a different protein architecture or by introducing site‐specific mutations, its conformational state can be shifted between CW and CCW. Together with ample functional and mutational data on chemotaxis receptors, there hence is overwhelming evidence that HAMP domains dynamically transition between CW and CCW, and that these transitions underpin signal transduction.

### 4.2 *The dynamics of α‐helical coiled coils*

HAMP domains widely recur in receptors at the junction between the transmembrane α helices and the intracellular effector moiety, that is, the DHp/CA domains in case of SHKs. Many receptors possess tandem arrays of several concatenated HAMP domains with the AS2 helices of the more upstream HAMP module merged with the AS1 helices of the more downstream HAMP module.^140^ Based on fundamental considerations of coiled‐coil geometry and structural data, two mechanistic models have been advanced for HAMP signal transduction. The gearbox model^139^ envisions that as part of the signaling process the AS1 and AS2 helices rotate by ~26° to transition between *a*–*d* and *x*‐*da* packing, akin to cogwheels in a motor. Within this model, the principal output mode that transmits to the effector would hence be a rotation of the AS2 helices. In the dynamic‐bundle model,^144^, ^145^ HAMP domains are deemed to be in equilibrium between tightly and loosely packed helical bundles that further differ in the relative angles and orientations of their constituent helices. Consequently, the principal outputs of the CW–CCW transition are helical rotation, pivoting (or, scissoring) and possibly changes in coiled‐coil register. As noted before,^141^, ^143^ the two models are not in contradiction but rather agree in key aspects, especially regarding the inherent equilibrium between two states and the generation of helix motions as the output signal. Taken together, HAMP domains convey, modulate and integrate signals traveling from extracellular/transmembrane sensors to the intracellular effectors of receptors. As seen above for SR,^128^ at least certain HAMP domains accept piston motions of the AS1 helices as input and convert them into helical rearrangements within their parallel four‐helix bundle.^84^, ^130^

Notably, the helical rearrangements generated as output by HAMP domains resemble those encountered in the LOV and BPhy photosensors treated above. The joint question for SHK signaling thus becomes, how are these conformational transitions propagated through the linker elements to the effector? The linkers between the sensors and effectors of homodimeric SHKs are parallel α‐helical coiled coils that directly feed into the DHp domain. Owing to the periodicity of coiled coils, see above, within the SHK family these linkers are of discrete lengths and exhibit alternating hydrophilic and hydrophobic residues.^12^, ^15^, ^36^, ^61^, ^102^ As recently summarized,^146^ helical bundles can undergo a series of principal transitions including dissociation/association, rotation, supercoiling (or, twisting), piston, and pivot motions. As several of these transitions can mutually compensate another, they commonly occur in concert rather than isolation. Hence, the principal transitions can be considered eigenmodes that in superposition make up a given conformational transition within a SHK linker. Piston motions and dissociation of individual helices within a bundle have been invoked for signaling by HAMP domains, see above. By contrast, within the linker that continues into the DHp domain, wholesale dissociation of the two helices or a piston shift of one helix relative to the other would incur large‐scale disruption of the coiled‐coil interface. While such transitions cannot be ruled out altogether, they appear unlikely. However, the existence of monomeric SHKs hints at the possibility of functionally relevant dimerization equilibria in SHKs.^6^ The principal conformational transitions to be considered within the parallel, homodimeric coiled‐coil linker are hence helical rotation, pivoting and supercoiling. Based on the above description of α‐helical coiled coils, it is apparent that these three transitions are in fact closely related and commonly occur in conjunction. As a particularly relevant example, an increase in (left‐handed) supercoiling entails a higher helix‐crossing angle, that is, leads to helix scissoring. Vice versa, such a pivot motion may increase the crossing angle between two helices and thereby promote their superhelical winding. Likewise, a right‐handed rotation of a (right‐handed) α helix, that is, a CCW rotation when viewed from the C terminus of the helix, has similar effects as the introduction of a stammer, that is, the omission of a residue within the helix.^133^ As described above, stammers can be accommodated in a parallel coiled coil by increased left‐handed supercoiling, that is, transitioning from the 7/2 to the 10/3 form. Hence, the at first glance disparate transitions of pivoting, rotation and supercoiling may in fact channel into conformationally equivalent states within the coiled coil.

Support for functionally relevant changes in helical supercoiling within the coiled‐coil linker derives from both biochemical data and structural analyses. In several systems, sequence and length variations of the linker implied its coiled‐coil structure and signal‐dependent changes in α‐helical supercoiling.^147^, ^148^, ^149^ For the present scope, ample data on the blue‐light‐responsive LOV histidine kinase YF1 are most relevant.^28^, ^60^, ^61^, ^150^ Variation of the linker between the LOV photosensor and the DHp domain in YF1 revealed a pronounced heptad, that is, seven‐residue, dependence of activity and response to blue light on linker length, indicative of the continuous coiled‐coil structure of the linker. SHK variants with 7·*n* residues in their linker, such as the original YF1, exhibited blue‐light‐repressed net kinase activity. Intriguingly, the addition of single residues, corresponding to a stutter within the coiled coil and giving rise to linkers with 7·*n* + 1 residues, sufficed for inversion of the blue‐light response; that is, the corresponding SHK variants were activated in their net kinase activity upon light absorption rather than inhibited. Put another way, YF1 variants with 7·*n* residues in their linkers assume their kinase‐active state K in darkness, whereas variants with 7·*n* + 1 residues only do so after blue‐light absorption. Notably, within an unperturbed α helix, a one‐residue difference, that is, between 7·*n* and 7·*n* + 1, corresponds to an angular difference of 100° around the helix axis. An increase in left‐handed supercoiling of the coiled coil would entail compensatory angular reorientation and move the 7·*n* + 1 to the 7·*n* position.^74^ Taken together, light‐induced conformational transitions within the LOV photosensor, see above, apparently propagate through the coiled‐coil linker as left‐handed supercoiling or torque; light is acting as a rotary switch.^61^

**Figure 7:**
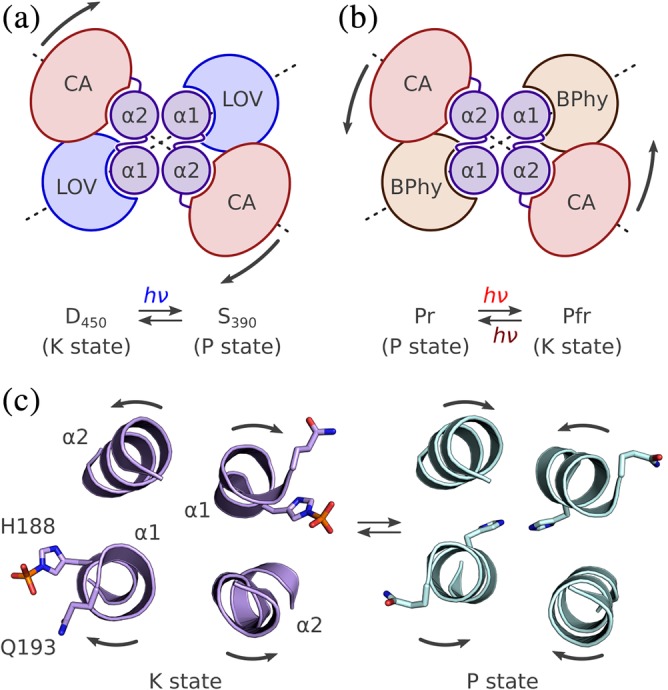
Structural basis of sensor histidine kinase regulation. (a) Blue‐light absorption triggers increased left‐handed supercoiling of the coiled‐coil linker of YF1 and thereby switches the receptor from the K to the P state. Viewed along the C_2_ axis of the receptor from C to N terminus, left‐handed supercoiling of the linker would translate into global left‐handed twist of the receptor (indicated by the arrows), as indeed determined by X‐ray solution scattering and molecular modeling.^76^ (b) In case of the *Deinococcus radiodurans* BPhy histidine kinase, solution scattering indicates that red light triggers a right‐handed twist of the receptor. The opposite handedness correlates with the transition from P to K state upon light absorption, rather than from K to P as in YF1. (c) Structural snapshots of *B. subtilis* DesK reveal at the molecular level how supercoiling and helical rearrangements channel into switching between the K and P states. Within the K state, the active‐site histidine (H188 in DesK) within helix α1 of the DHp domain is solvent‐exposed and thereby capable of catalyzing autophosphorylation and phosphotransfer to the response regulator. Within the P state, the histidine is sequestered into the DHp interior, and residues engaged in the phosphatase reaction are instead moved into position. The arrows denote the helical rearrangements that interconvert the K and P states. The molecular graphics are based on the structures of DesK in its autophosphorylated and phosphatase states, respectively^152^ (PDB 5IUM and 5IUN)

Atomically resolved information on the linker and its signal‐induced conformational changes is scarce because the vast majority of structural data have been obtained on SHK fragments that entirely lack the linker. Even where resolved in the structure, the linker is often compromised by truncation of the sensor or effector modules, potentially causing fraying of the linker termini.^151^, ^152^ In addition, the assignment of a given truncated structure to a specific functional state of the SHK can be challenging, thus further complicating the analysis. To date, the prototypical homodimeric transmembrane SHKs have eluded structural elucidation at full length. Certain noncanonical SHKs aside,^6^, ^26^ structural information at (or, near) full length is only available for VicK^153^ from *Streptococcus mutans* and for the engineered LOV histidine kinase YF1 in its dark‐adapted, kinase‐active K state.^60^, ^61^ Within both receptors, the sensor module comprises a PAS/LOV homodimer that connects to the effector module via a short coiled coil. Notably, the coiled coil forms continuous helices with the DHp domain, which benefits downstream transmission of the conformational signals discussed above. To fully unravel signal‐dependent conformational transitions of the linker, one would ideally require atomically resolved structural information on the same SHK for different functional states. Although structural data of that type are not available yet, the conformational transitions that the YF1 SHK undergoes upon blue‐light absorption were recently charted by EPR spectroscopy^74^ and X‐ray solution diffraction.^75^, ^76^ The time‐resolved scattering data revealed a biphasic response of full‐length YF1 to light absorption. A fast phase, rate‐limited by photochemical events within the LOV photosensor, see above, occurs on the microsecond scale and is followed by a much slower phase on the millisecond scale. To obtain structural information on these transitions, molecular dynamics simulations were based on the dark‐adapted YF1 structure and evaluated against the scattering data. This analysis indicated that within the fast phase after light absorption, the YF1 receptor undergoes a global twist by about 10–20^0^ in left‐handed direction when viewed from the N‐ to the C‐terminus of the molecule (Figure 7a), accompanied by a straightening of the entire receptor. Although the resolution of the scattering data is insufficient to atomically resolve the structure of the linker, the detected larger‐scale conformational changes would be accounted for by left‐handed supercoiling of the coiled‐coil linker. Intriguingly, left‐handed supercoiling upon light absorption is exactly the conformational mode that the biochemical data on YF1 linker variants suggest. Taken together, the functional and structural data thus both implicate light‐induced left‐handed supercoiling of the linker coiled coil as the principal mode of signal transduction from the sensor to the effector module.

A compellingly similar scenario emerges for the BPhy SHK from *D. radiodurans*, for which structures of the photosensory core module are available in both the Pr and Pfr states,^99^, ^100^ see above. Based on this information and X‐ray solution scattering experiments,^154^ the overall conformational transitions in the full‐length receptor upon going from Pr to Pfr were modeled by molecular dynamics. Strikingly, this analysis revealed a light‐induced global twist of the receptor as well, albeit of larger extent (around 50^0^) and of opposite direction, that is, a right‐handed mode when viewed from N‐ to C‐terminus (Figure 7b). If these data are to be reconciled with those acquired for YF1, one must explain the opposite direction of twist upon light exposure. Although detailed biochemical data on *Dr*BPhy are lamentably scarce, the light‐dependent downstream effect it exerts on pigmentation of *D. radiodurans* implies that the receptor acts as a red‐light‐activated SHK.^90^ This finding contrasts with YF1, which operates as a blue‐light‐repressed net kinase and thus rationalizes the opposite directionality of light‐induced twisting. Taken together, the transition from kinase‐active state K to phosphatase‐active state P, as occurs in YF1 upon light exposure, can tentatively be associated with a left‐handed receptor twist, and that of the inverse transition from P to K with a right‐handed twist.

Sequence analyses imply that coiled‐coil linkers abound in SHKs and other receptor classes, and by extension, similar signaling mechanisms might be at play. Among the photoreceptors, characteristic heptad periodicities of the linker length, indicative of coiled coils, have been reported for several families of LOV and BPhy receptors.^12^, ^36^, ^61^, ^102^ Similar sequence signatures recur in the linkers between consecutive (photo)sensor modules which argues that coiled coils are also instrumental for the integration of multiple signals. This notion is implied by the tandem arrangements of regularly spaced PAS^15^ and GAF domains, for example, in cyanobacteriochromes,^155^ evident in many receptors,^56^ and it has been borne out experimentally in the engineered SHK YHF.^15^ Derived from YF1, YHF combines a LOV with a heme‐binding PAS sensor and responds to the signals blue light and molecular oxygen in positive cooperative manner. While the molecular bases of signal integration in YHF and related receptors await elucidation, they could hinge on conformational transitions of coiled‐coil assemblies akin to the ones discussed presently.

## 5 EFFECTOR OUTPUT

The structure of SHK effector modules and their conformational transitions underpinning function have been the subject of recent, excellent review articles.^13^, ^156^, ^157^, ^158^, ^159^ Rather than revisiting in depth the vast body of structural data on SHKs, this treatise will focus on key aspects as they pertain to the present scope of photoreceptor histidine kinases. The above sections illustrate how light signals are absorbed, converted into protein conformational changes, and channeled into the homodimeric α‐helical bundle that constitutes the SHK linker. In a nutshell, signal induces distortion and displacement of the linker helices, with left‐handed supercoiling emerging as the most relevant principal mode, at least for photoreceptor histidine kinases. The key question then is how helical supercoiling can alter the equilibrium between the functional K and P states of the DHp/CA effector.

Among all the SHKs characterized structurally, the most complete and hence, most informative data sets to date have been acquired for HK853 from *Thermotoga maritima* 7, 160, 161, 162 and DesK from *B. subtilis*.^151^, ^152^, ^157^, ^159^ High‐resolution data, available for different functional and structural states of both HK853 and DesK, illustrate at the molecular level the conformational transitions underlying kinase and phosphatase action of the SHK. The structural data are best developed for DesK, and hence the following discussion will be primarily based on this SHK. For an authoritative description of these processes in DesK, the reader is referred to Figure 6 of Trajtenberg et al.^152^ Independent of the functional state, the DHp domain consists of two long helices, denoted α1 and α2, that within the homodimeric receptor form an antiparallel heterotetrameric coiled coil of the $A_{2}{\bar{B}}_{2}$ type.^160^ The helices α1 and α2 are connected by short hairpin loops in right‐handed or left‐handed topology.^163^ Depending on the handedness, the active‐site histidine within helix α1 is positioned either in proximity to the catalytic domain CA of the same monomer which results in autophosphorylation in *cis*, or near CA of the sister monomer which promotes autophosphorylation in *trans*.^13^, ^163^ The globular CA domain itself is appended to the C terminus of α2 via an unstructured connector and can display widely different placements and angular orientations relative to the DHp domain.^13^ Despite this structural variability, the kinase‐active K state of SHKs is characterized by conformations of the antiparallel heterotetrameric DHp coiled coil that position the catalytically active histidine, H188 in DesK, in a solvent‐exposed, outward‐facing orientation (Figure 7c). The active‐site histidine is thus accessible for interactions with the CA domain during autophosphorylation and the RR during phosphotransfer, respectively. Several SHK structures in their K states, for example, those of VicK and YF1, recurrently exhibit kinks that arise in different places within the DHp domain but invariably break the C_2_ symmetry of the receptor.^60^, ^152^, ^153^ Given the prevalence of these bends within the K state, they might be functionally relevant.^157^ Structural snapshots reveal that the K state can readily accommodate both the autophosphorylation and phosphotransfer reactions.^152^, ^157^ During the autophosphorylation reaction, the CA domain docks onto the DHp domain such that the γ phosphoryl group of its bound ATP factor is in line with the active‐site histidine. To enable nucleophilic attack, the histidine forms a catalytic dyad which the immediately succeeding residue, D189 in DesK. Upon phosphorylation, the CA domain likely detaches, and the phosphorylated histidine sidechain is thought to adopt a different rotamer.^152^ To allow phosphotransfer, the RR binds to the lower half of the DHp domain in an orientation that places its conserved aspartyl residue such that it can accept the phosphoryl group from the SHK histidine. Notably, the interaction between DHp and RR is highly specific, thus structurally insulating different TCSs from another and reducing cross‐talk.^164^, ^165^, ^166^

As revealed by a series of DesK structures,^152^ within the P state the bottom half of the DHp domain remains largely invariant, thus preserving the binding site for the RR which associates in a very similar orientation as in the K state. By contrast, the DHp upper part is reconfigured in the P state relative to the K state in two principal regards (Figure 7c,d). First, the α1 helices that N‐terminally connect to the linker coiled coil, see above, are inclined relative to each other and to the α2 helices at greater angles. When evaluated over residues 185–193 of helix α1, the crossing angle in the K state amounts to around 22° and that in the P state to 34°. Second, all four DHp helices are slightly rotated in concert around their longitudinal axes (Figure 7c,d). These rearrangements position the histidine sidechain toward the inside of the DHp helix bundle within the P state and effectively sequester it from the solvent. Concomitantly, the sidechain of residue Q193 (in DesK) rotates into place to coordinate a water molecule, which in turn can hydrolyze the phospho‐aspartyl anhydride bond within the RR. Notably, biochemical data on SHKs of the Pfam^56^ HisKA_3 class, that DesK belongs to, had pinpointed this residue as part of a conserved HDxxxQ motif that is essential for the phosphatase reaction.^167^

Although the structural information for other SHKs is not nearly as rich as for DesK and HK853, the fundamental catalysis mechanisms and the conformational equilibrium between the K and P states elucidated in the paradigm systems appear compatible with SHK signaling in general. Applied to YF1, the following scenario emerges: In the K state (adopted in darkness), the active‐site histidine 161 would point outwards and, in concert with E162, mediate autophosphorylation and subsequent phosphotransfer to the RR FixJ. Within the P state, H161 would be sequestered inside the DHp interior and Q165 moved in place to allow hydrolysis. Notably, as members of the Pfam HisKA family, YF1 and HK853 feature a slightly different phosphatase consensus motif of HExxζ, where ζ denotes a hydrophilic residue, for example, T, N, or Q.^56^ Based on the above‐described findings for DesK, in the P state of YF1 the DHp α1 helices are expected to be slightly rotated in left‐handed manner and to cross at a somewhat increased angle. Intriguingly, these structural transitions are fully compatible with the blue‐light‐triggered sequence of events in the linker of YF1, as suggested by both solution scattering and biochemical data, see above. Left‐handed supercoiling of the linker would directly promote productive rearrangements of the DHp α1 helices and thereby drive the transition from the K state, prevailing in darkness, to the P state under blue light. Moreover, the concerted rotation and tilting of the DHp helices when going from the K to the P state change the angular orientation of the effector relative to the linker and sensor moieties. This transition would amount to a global left‐handed twist (when viewed from N to C terminus of the SHK) which is indeed observed in the SAXS data (Figure 7a). Combined with information derived from the DesK and HK853 systems, the available structural and functional data on YF1 enable the construction of a full molecular trajectory of events induced by blue light and culminating in SHK regulation, as shown in Movie S1. Given the similarity of the structural outputs generated by LOV, BPhy and rhodopsin sensor modules upon light absorption, that is, helical rearrangements within a parallel homodimeric coiled coil, discussed above, it appears likely that similar signaling trajectories apply to these photoreceptor histidine kinases, too.

## 6 CONCLUSION

In contrast to the prevalent light‐inert, transmembrane SHKs, photoreceptor histidine kinases are mostly soluble proteins, which greatly benefits detailed mechanistic studies of signal detection, processing and transduction. The signal, light of suitable color, is known and can be applied or withdrawn easily, thereby facilitating the interrogation of both the kinase‐active K and the phosphatase‐active P states, even in time‐resolved manner. Photoreceptor histidine kinases hence serve as relevant and experimentally tractable paradigms for TCS signaling. The current recapitulation of findings on receptors coupled to light‐oxygen‐voltage, bacteriophytochrome and microbial rhodopsin photosensor units arrives at a detailed and astonishingly uniform molecular view of signal transduction. Notwithstanding substantial disparity in protein architecture, the three photosensor classes invariably decode incoming light signals into similar and simple outgoing conformational modes that take the form of repositioned α helices, chiefly through pivot and rotary movements, or a combination thereof. These conformational perturbations translate to the ensuing linker segments where they converge to modulate coiled‐coil structure, in particular the degree of left‐handed superhelical winding. Changes in superhelical structure readily propagate to the effector module where they allosterically regulate the SHK. Allostery, as advanced by Monod, Wyman and Changeux,^10^ posits that signal stabilizes or destabilizes the inherent (meta)stable states of a receptor, thereby modulating the dynamic equilibrium between them, without much modifying the nature of these states. Ground‐breaking structural insights, especially from the DesK^151^, ^152^, ^157^, ^159^ and HK853^7^, ^160^, ^161^, ^162^ model systems, reveal how allostery plays out at the molecular in level in SHKs. Key to the transition from the K to the P state are rearrangements of the DHp α1 helices that bring about changes in the solvent accessibility of residues engaged in catalyzing the forward kinase and reverse phosphatase reactions. The active site is thus reconfigured and converted from kinase to phosphatase activity.

Despite its largely invariant structure, the effector module of SHKs apparently tolerates sensory input from highly disparate sensor units, ranging from photosensors covered here to diverse and versatile sensors for other signals.^56^ Strikingly, the conformational changes in the SHK that underpin signal‐dependent regulation are often subtle, with a spatial extent of a few ångströms only. It is tantalizing to speculate that the signaling strategies that presently emerge for photoreceptor histidine kinases more generally also pertain to other SHKs, including transmembrane receptors. This notion is not least supported by the exchangeability of certain sensor units in SHKs.^29^, ^61^, ^168^ The underlying signaling mechanisms may hence be widely shared among SHKs or can at least be co‐opted. An improved understanding of these mechanisms also stands to inform the engineering of novel photoreceptors^14^, ^169^ to serve as efficient paradigms in the study of signal transduction and as light‐gated actuators in optogenetics. Notably, the photosensor units discussed here and related ones might be leveraged for widely regulating by light the activity of homodimeric target proteins, especially if they comprise parallel α‐helical coiled coils.

## Supporting information


**Supplementary Movie 1** The animation describes the likely sequence of molecular events in the light‐oxygen‐voltage histidine kinase YF1 after blue‐light absorption.
